# The Sheffield Medical Journal

**Published:** 1892-12

**Authors:** 


*The Sheffield Medical Journal.* A Quarterly Review of the Medical Sciences for Yorkshire and adjoining Counties. Vol. I. Part I. Pp. 80. Sheffield: PAWSON and BRAILSFORD. October, 1892.

We are glad to welcome this, the latest, addition to the now goodly list of provincial medical journals, and are gratified to have so sturdy and promising an infant in our Exchange List. These local journals cannot fail to be of great benefit to the profession in their neighbourhood: the time and labour given to them by their editors and workers, although sometimes apparently unprofitable and occasionally thankless, must tend to the promotion of literary talent, and to the production of useful work. We need have no fear that a process of literary decentralisation will be carried too far: our metropolitan journals have no misgivings as to their provincial contemporaries. The local medical journal is becoming a necessity, inasmuch as the metropolitans are not able to keep pace with the supply, and it will be an addition to, rather than a displacement of, existing literature. It is often said that we have too much to read, and that life is too short for all the literature provided; but nevertheless it is remarkable to note that it is only the busiest of men who find time to keep up with current medical literature. We congratulate the editor and his associates on the appearance and the contents of their first number, and wish them that amount of support from the profession of the district which their efforts clearly deserve.

